# Editorial: Acquired Heart Disease in Children: Pathogenesis, Diagnosis and Management

**DOI:** 10.3389/fped.2021.725670

**Published:** 2021-07-15

**Authors:** Ying Liao, Hongfang Jin, Xupei Huang, Fangqi Gong, Lijun Fu

**Affiliations:** ^1^Department of Pediatrics, Peking University First Hospital, Beijing, China; ^2^Department of Biomedical Science, Charles E. Schmidt College of Medicine, Florida Atlantic University, Boca Raton, FL, United States; ^3^Department of Cardiology, National Clinical Research Center for Child Health, Children's Hospital, Zhejiang University School of Medicine, Hangzhou, China; ^4^Department of Cardiology, Shanghai Children's Medical Center, Shanghai Jiao Tong University School of Medicine, Shanghai, China

**Keywords:** myocarditis, pericarditis, cardiomyopathy, pathogenesis, management

Myocarditis, pericarditis, and cardiomyopathy are important acquired heart diseases during childhood. Under the Research Topic “Acquired Heart Disease in Children: Pathogenesis, Diagnosis and Management,” totally seven literatures focused on the above diseases and shed light on the pathogenesis, diagnosis and treatment of the diseases, attracting large numbers of readers, and stimulating their thinking in the future studies on these diseases.

## Myocarditis

The diagnosis and treatment of myocarditis, especially acute fulminant myocarditis (AFM), in pediatric population are challenging. Three articles in this Topic reported the research advance in pediatric AFM, although it is unclear why children with AFM manifest such critical symptoms and rapid progression, abnormal immune response triggered by certain pathogens in susceptible individuals may be considered as possible mechanisms (1). To investigate the potential pathogenesis of AFM, Liu et al. analyzed the profiles of long non-coding RNAs (lncRNAs) and mRNA in the leukocytes of pediatric patients with AFM as well as controls using microarrays and found that the expressions of lncRNAs and mRNAs were distinctly different between the two groups of the subjects. Further analysis on the potential biological function and the molecular interactions of these differentially expressed genes revealed that immune processes such as T cell activation and several signaling pathways related to immune activation may take part in the pathogenic process of AFM. Furthermore, the study showed that most differentially expressed lncRNAs were related to the target genes that were adjacent to them. Although the functional prediction still needs to be verified, these results may provide important clues on the immune mechanisms for AFM. Early diagnosis and appropriate evaluation for patients' condition are pivotal aspects in the management of pediatric AFM. Lv et al. reviewed 20 pediatric patients with AFM and summarized their clinical features. Particular attention was paid to the value of cardiovascular magnetic resonance (CMR) in the diagnosis as well as follow-up of AFM in this study. Abnormal findings of CMR, mainly including high signal in T2-weighted image and late gadolinium enhancement, were determined in 80% of the cases. Another study from Zhu et al. retrospectively analyzed the alert symptoms and other manifestations of 23 pediatric patients with AFM. The above two studies showed that extracardiac symptoms were the most common initial manifestations of their patients with AFM, which was also consistent with the previous studies (2). Therefore, it is suggested that hypoperfusion signs with abdominal pain, vomiting, dizziness, or/and convulsions may be the alert of AFM in children. Besides, treatment for children with AFM included intravenous immunoglobulin (IVIG), glucocorticoids and the supportive therapies such as continuous renal replacement therapy, extracorporeal membrane oxygenation, and temporary pacemakers according to the two studies.

Although it is believed that the pathogenesis of myocarditis may involve abnormal immune reaction to the pathogen, the present views on immunosuppressive therapy with steroids or immunomodulatory therapy with IVIG for treating myocarditis in children are still controversial (3, 4). To evaluate the therapeutic response to corticosteroids and IVIG in pediatric myocarditis, Li et al. conducted a meta-analysis on the topic. The results supported that IVIG was effective in enhancing the impaired left ventricular ejection fraction (LVEF) as well as survival for children with myocarditis; while corticosteroids didn't show an efficacy advantage over the conservative treatment. The authors also called for large sample size, randomized controlled trials because of the limited numbers of associated studies on the topic.

## Pericarditis

Recurrence pericarditis (RP) stands for the recurrence of acute pericarditis after a recorded first course with a symptom-free interval of at least 4–6 weeks (5). Tombetti et al. reviewed the present advances in etiology, pathogenesis, diagnosis, management, and prognosis of RP in children and adolescents. Tuberculosis is still the commonest cause of RP worldwide, while 70% of children with RP were attributed to an idiopathic origin in developed countries. It was recommended that the genetic factors should be paid great attention in children with RP when certain warning signals existed. As for the prognosis, deaths related to idiopathic RP were rare, and frequent recurrence, cardiac tamponade, pericardial constriction, and myocardial involvement were the most concerned conditions. They also summarized some risk factors of the recurrence and complications, which were helpful for clinical practice. Anti-IL1 therapy was highlighted in the management of RP based on an accurate diagnosis and a reasonable treatment algorithm.

## Cardiomyopathy

According to the latest scientific statement from the American Heart Association (AHA) on classification of cardiomyopathy in children, the morphofunctional phenotype is still the primary hierarchy in the diagnostic system, including dilated, hypertrophic, restrictive, non-compaction, and arrhythmogenic cardiomyopathies (6). In this Research Topic, Guo et al. evaluated a series of drug-related receptors in pediatric patients suffering from dilated cardiomyopathy (DCM). It is highly valuable that they analyzed the drug-related receptors on left ventricular tissue from children with idiopathic DCM who underwent heart transplantations, from the controls (heart donors) and from adult DCM. As a result, the difference in distributions of drug-related receptors on heart tissue provided significant clues to make a specific medication regimen for pediatric patients. Another article from Bai et al. focused on the genetic mechanisms for non-compaction of ventricular myocardium (NVM). They discovered a *de novo MTUS1* mutation from a rare family with NVM and found that the mutation can reduce the stability of microtubules and increase the polarity of cell through the Rac1/Cdc42 signaling pathway in *in vitro* studies. However, the role of this mutation in *MTUS1* gene in the pathogenesis of NVM still merits further studies.

In summary, the articles from this Research Topic showed the latest advances in the above acquired heart diseases in children. The discoveries would not only deepen the understanding of the mechanisms, but also bring important improvement to clinical practice. However, there are still a lot of issues in this field, such as the pathogenesis of pediatric myocarditis, cardiomyopathy as well as pericarditis, and the biomarker-based individualized therapy for different categories of cardiomyopathies, need further investigation.

## Author Contributions

HJ organized the Research Topic and revised the editorial. YL wrote the draft of this editorial. XH, FG, and LF reviewed the Research Topic articles and revised the manuscript. All authors listed have made a substantial, direct and intellectual contribution to the work, and approved it for publication.

## Conflict of Interest

The authors declare that the research was conducted in the absence of any commercial or financial relationships that could be construed as a potential conflict of interest.
